# Successful Antiviral Treatment with Direct-Acting Antivirals for Hepatitis C Virus Infection during Peritransplant Period in a Kidney Transplant Recipient

**DOI:** 10.1155/2021/1948560

**Published:** 2021-12-11

**Authors:** Giovanni Varotti, Ferdinando Dodi, Ernesto Paoletti, Andrea Bruno, Iris Fontana

**Affiliations:** ^1^Kidney Transplant Unit, San Martino University Hospital, Genoa, Italy; ^2^Department of Infectious Diseases, San Martino University Hospital, Genoa, Italy; ^3^Department of Nephrology, San Martino University Hospital, Genoa, Italy

## Abstract

*Introduction*. Hepatitis C virus (HCV) infection continues to represent a poor prognostic factor in kidney transplant (KTx) patients. New direct-acting antiviral agents (DAA) have dramatically changed the therapy management for HCV, showing promising results in terms of sustained virologic response. Timing for DAA therapy in HCV positive kidney waitlist patients continues to be controversial, and caution is recommended due to the potential difficult immunosuppressant dose adjustments, particularly in the early posttransplant period. We report a case of a KTx performed during antiviral DAA therapy. *Report of Case*. Patient was a 44-year-old man suffering from chronic HCV hepatitis associated with end-stage kidney disease (ESRD), waitlisted for a second KTx as a sensitized patient (panel-reactive antibody peak 85%) in March 2019. Four months later, antiviral DAA therapy was started (glecaprevir/pibrentasvir 300 mg/120 mg daily, for 8 weeks). After 30 days, a left kidney was offered and, given the good compatibility, we decided to proceed with KTx without discontinuing the DAA therapy. A standard straightforward kidney transplant was performed. Immunosuppression included thymoglobulin and prednisone for induction and tacrolimus and mycophenolate for maintenance. After a transient delay graft function, creatinine levels progressively decreased. From postoperative day 3, tacrolimus reached target levels and remained stable. No episodes of acute rejection occurred. The 8-week DAA therapy was carried out without interruption. All HCV-RNA level controls resulted undetectable. On postoperative day 15, the patient was discharged and remains in healthy condition with normal renal function and HCV negative after 18 months of follow-up. *Discussion*. In this case, DAA therapy during the perioperative KTx period was well tolerated and effective. If confirmed, patients should not necessarily be suspended from the waiting list during DAA therapy for HCV eradication.

## 1. Introduction

Hepatitis C virus (HCV) infection is associated with an increased risk of morbidity and mortality in end-stage kidney disease (ESRD) and in kidney transplant (KTx) patients [1].

The new antiviral direct-acting agents (DAA) have dramatically changed the management of HCV therapy by showing optimal safety and efficacy.

Different types of DAA have been proposed for HCV eradication in ESRD and KTx patients with evidence of a high-sustained virologic response (>95%) [2].

Timing for DAA therapy in HCV-positive kidney waitlist patients continues to be controversial. Although no specific limitation or contraindication is described in international guidelines [3, 4] and many studies have indicated that DAAs can be safety administered after renal transplantation, some caution is recommended due to the potential difficult dose adjustment with immunosuppressants, especially in the early posttransplant period [5].

We report a case of a KTx performed during the antiviral DAA therapy.

## 2. Case Report

The patient was a 44-year-old man suffering from chronic HCV Hepatitis (genotype 1b) associated with ESRD secondary to malignant hypertension. The patient first had a KTx in 2005 and had been on hemodialysis since 2016. Liver function was normal, and the ultrasound elastography showed light liver fibrosis (METAVIR score F1). In March 2019, the patient was listed for a second KTx as a sensitized patient (panel-reactive antibody peak 85%). Four months later, antiviral DAA therapy was started (glecaprevir/pibrentasvir 300 mg/120 mg daily, for 8 weeks) [4]. HCV-RNA viral load at the initiation of DAA therapy was 3 × 10^6^ IU/L. After 30 days, a left kidney from a 63-year-old deceased donor was offered. Given the good compatibility (no mismatches), we decided to proceed with the KTx without discontinuing the DAA therapy. A standard straightforward kidney transplant was performed.

Immunosuppression included thymoglobulin and prednisone for induction and tacrolimus and mycophenolate for maintenance.

**Figure 1:**
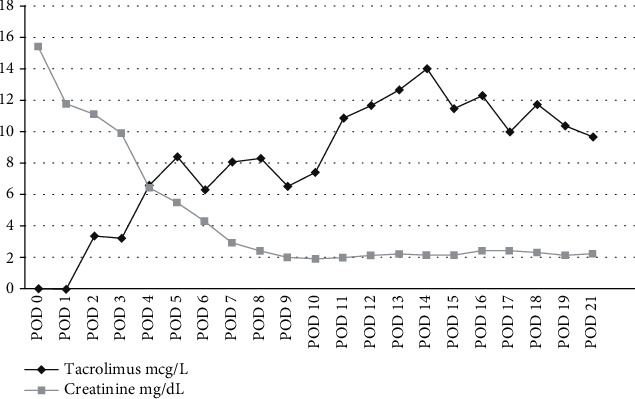
Posttransplant levels of tacrolimus and serum creatinine.

After a transient delay graft function, creatinine levels progressively decreased, and from postoperative day 3, tacrolimus reached target levels and remained stable in the following period (Figure 1). No episodes of acute rejection occurred, and the 8-week DAA therapy was carried out without interruption. All HCV-RNA viral load controls after KTx resulted undetectable. On postoperative day 15, the patient was discharged and remains in healthy condition with normal renal function and was HCV-negative after 24 months of follow-up.

## 3. Discussion

To the best of our knowledge, there are no other cases reporting KTx being performed during DAA therapy.

In accordance with the international HCV guidelines [3, 4], the duration of the DAA therapy was 8 weeks. In fact, recent evidences have shown that an 8-week treatment of glecaprevir/pibrentasvir in naive patients without cirrhosis (any genotype) can achieve optimal sustained virologic response rates, not inferior to those achieved with a 12-week treatment, as reported by previous studies [6–8].

Moreover, the best timing for DAA therapy continues to be debated because while DAA therapy performed before transplant avoids risk of HCV progression, the idea of eradicating the HCV after the Tx has the advantage of expanding the pool of donors to those HCV-positive.

As a consequence, there is a trend to use DAA post-Tx in centers with a high volume of HCV-positive donors and vice versa [9].

In the specific case of HCV-positive wait-listed patients, there are two options: to start DAA therapy prior to transplant, requiring the patient to be temporarily suspended from the waiting list, and consequently losing the chance of a possible transplant. Alternatively, DAA therapy can be postponed for at least six months post-KTx, increasing, however, the risk of HCV-related complications [4–6]. Although there are no specific contraindications to start DAA early post-KTx, some limitations can occur with respect to the potential interaction with immunosuppressant drugs (Table 1). In particular, coadministration of glecaprevir/pibrentasvir with systemic tacrolimus is associated with an increased tacrolimus Cmax and AUC. As a consequence, its use is recommended with caution and with strict therapeutic blood monitoring.

In our case, as a result of optimal donor/recipient immunological matching, we decided to proceed with the KTx, and following a strict monitoring of the tacrolimus plasmatic concentration, we experienced no particular difficulties in the management of immunosuppression dosages (Figure 1).

If our findings are confirmed, patients should not necessarily be suspended from waiting lists during DAA therapy as it should not be considered a contraindication for KTx.

## Figures and Tables

**Table 1:** Degree of safety of coadministration of glecaprevir/pibrentasvir and immunosuppressants (red: do not coadminister; orange: potential interaction; yellow: potential weak interaction; green: no interaction expected) [10].

	Glecaprevir/pibrentasvir
Tacrolimus	Orange
Mycophenolate	Green
Methylprednisone	Green
Prednisone	Green

## Data Availability

The data used to support the findings of this study are included within the article.

## Abbreviations

HCV: Hepatitis C virus
ESRD: End-stage renal disease
KTx: Kidney transplantation
DAA: Direct-acting antiviral agent.
